# Investigations of Shape, Material and Excitation Wavelength Effects on Field Enhancement in SERS Advanced Tips

**DOI:** 10.3390/nano11010237

**Published:** 2021-01-18

**Authors:** Yaakov Mandelbaum, Raz Mottes, Zeev Zalevsky, David Zitoun, Avi Karsenty

**Affiliations:** 1 Advanced Laboratory of Electro-Optics (ALEO), Department of Applied Physics/Electro-Optics Engineering, Jerusalem College of Technology, Jerusalem 9116001, Israel; ymandelb@g.jct.ac.il (Y.M.); rm1995ex@gmail.com (R.M.); 2 Faculty of Engineering, Bar-Ilan University, Ramat Gan 5290002, Israel; Zeev.Zalevsky@biu.ac.il; 3 Nanotechnology Center, Bar-Ilan University, Ramat Gan 5290002, Israel; 4 Faculty of Exact Science, Department of Chemistry, Bar-Ilan University, Ramat Gan 5290002, Israel; David.Zitoun@biu.ac.il; 5 Nanotechnology Center for Research and Education, Jerusalem College of Technology, Jerusalem 9116001, Israel

**Keywords:** TERS, SERS, nano-cones, nano-cavities, plasmon, numerical, analytical model

## Abstract

This article, a part of the larger research project of Surface-Enhanced Raman Scattering (SERS), describes an advanced study focusing on the shapes and materials of Tip-Enhanced Raman Scattering (TERS) designated to serve as part of a novel imager device. The initial aim was to define the optimal shape of the “probe”: tip or cavity, round or sharp. The investigations focused on the effect of shape (hemi-sphere, hemispheroid, ellipsoidal cavity, ellipsoidal rod, nano-cone), and the effect of material (Ag, Au, Al) on enhancement, as well as the effect of excitation wavelengths on the electric field. Complementary results were collected: numerical simulations consolidated with analytical models, based on solid assumptions. Preliminary experimental results of fabrication and structural characterization are also presented. Thorough analyses were performed around critical parameters, such as the plasmonic metal—Silver, Aluminium or Gold—using Rakic model, the tip geometry—sphere, spheroid, ellipsoid, nano-cone, nano-shell, rod, cavity—and the geometry of the plasmonic array: cross-talk in multiple nanostructures. These combined outcomes result in an optimized TERS design for a large number of applications.

## 1. Introduction

The need for the development of real-time sensors, capable of monitoring continuous flow reactions and phenomena, became one of the next challenging frontiers to reach in chemical sensing. This is why imaging sensors, capable of recording and reporting spatial variations in real time, are more than desirable. The Surface Enhanced Raman Scattering (SERS) method, capable of chemical sensing, is used either on chemicals which are adsorbed on a particular substrate, by scanning with a sharp metallic tip [1,2], or by dispersing metallic nano-particles into the solution [3]. The first and second methods preclude real time detection. The first method is not capable of self-refreshing, and the second method due to lengthy scanning times; the third method is not position specific. Thus, the development of an imaging sensor which is both dynamic and has specificity in space is more than justifiable. It is here envisioned as an array of SERS nanostructures, tips or cavities, with the capability of fulfilling these demands.

Fleischmann et al. first observed SERS from pyridine adsorbed on electrochemically roughened silver in 1973 [4]. A few years later, several research teams working in parallel, arrived at the same observations, noting that the scattering species concentration could not explain the enhanced signal. In fact, each team suggested a different approach to explain the observed phenomenon, and the explanatory mechanisms they proposed for the SERS effect are still accepted today. Jeanmaire and Van Duyne [5] proposed a theoretical mechanism by which Raman signals are amplified by an electric field enhancement near a metallic surface.

When a substrate is impinged by incident light source, the phenomenon generates an excitation of the localized surface plasmons. The electric field enhancement produced near the surface, is maximized in the resonant condition when the frequency of the incident light is equal to the surface plasmon frequency. Thus, Raman signals’ intensity for the adsorbates is increased due to electric field enhancement near the substrate. The size, shape and material of the nano-particles determine the electromagnetic enhancement of the SERS, which is theoretically calculated in order to enable factor values in the range of ~10^10^–10^11^ [6]. This enhancement factor [EF] can be approximated by the magnitude of the localized electromagnetic field to the fourth power (the *E*^4^ approximation approach will be further discussed in this article).

While Jeanmaire and Van Duyne [5] proposed an explanation based on an electromagnetic effect, Albrecht and Creighton [7] proposed an alternative explanation based on charge-transfer effect. Raman spectrum peaks are enhanced due to intermolecular and intramolecular charge transfers. The high-intensity charge transfers from the metal surface with wide band to the adsorbing species causing great enhancement for species adsorbing the metal surface [8]. Because surface plasmon appears only in metal surface, with near-zero band gaps, the effect of Raman resonance enhancement is dominant in SERS for species on small nanoclusters with considerable band gaps. Although the charge-transfer effect explanation is less accepted than the electromagnetic effect, both effects can probably occur together for metal surfaces [9]. At the end, Ritchie predicted the surface plasmon’s existence years before [10].

The methods to perform and prepare SERS measurements have progressed with time, moving from electrochemically roughened silver [9], distribution of metal nano-particles on the surface [11], lithography [12], porous silicon support [13,14], and two-dimensional silicon nano-pillars embedded with silver [15]. The most common method consists today of liquid sample deposition onto a silicon or glass surface, with a nanostructured noble metal surface. The enhancement phenomenon is intensely affected by the geometry (both shape and size) of the metal nano-particles, since the ratio of absorption and scattering events are influenced by these factors [16,17]. According to Bao et al., it may be an ideal particles size and an ideal surface thickness as a function of each particular experiment [18]. For example, while the excitation of non-radiative multipoles is a result of exceptionally large particles, the loss of electrical conductance, and as a consequence the lack of field enhancement, is due to extremely small particles. For particles sharing only the size of a few atoms, there must be a large collection of electrons to oscillate together, since there is no defined plasmon [19]. Higher-order transitions cause the enhancement’s overall decrease in efficiency, since the dipole transition leads to Raman scattering. Both high uniformity and field enhancement define ideal SERS substrates, which are fabricated on wafer scale with label-free super resolution microscopy. Such a resolution was proven adequate, when using SERS signal’s fluctuations on such uniform and high-performance plasmonic meta-surfaces [20].

With the large development of SERS usage, and since a vast amount of literature became available, several publications focused in the last two decades on reviewing specific SERS sub-domains. While Pamela Mosier-Boss reviewed the substrates for chemical sensing [21], additional summarizing studies focused on substrates and analytes [22]. Others focused also on Surface-Enhanced Resonance Raman Scattering (SERRS), and on possible applications [23]. Van Duyne and other pioneers in the field presented a large perspective on the present and future achievements in SERS, both in the past [24] and more recently [25]. However, large-scale and methodical analysis of Tip-Enhanced Raman Spectroscopy (TERS) has not yet presented, while combining numerical and analytical complementary analyses. This is why this current study largely focuses, among others, on the investigations of TERS effects of shape, material and excitation wavelength on field enhancement (FE).

As part of the Surface-Enhanced Raman Spectroscopy (SERS) technique, one can find a more accurate approach entitled Tip-Enhanced Raman Spectroscopy (TERS). TERS is the combination of a scanning probe microscope and a plasmonic metal tip, and its strongest point, aside from its high chemical sensitivity, is the high spatial resolution (beyond the diffraction limit), and imaging it can provide information for data analysis. In this technique, Raman scattering enhancement occurs only at the extremity of a near atomically sharp pin, usually coated with gold [26]. In SERS spectroscopy, there are two limitations:

1. The signal is produced by the sum of a large number of molecules.
2. The resolution is limited to Abbe limit, which is half the wavelength of the incident light.

TERS overcomes these limitations by sampling only a small number of molecules near the tip, which consists of a few tens of nanometers. There are basically two kinds of TERS, which are generally accepted by the TERS community:

1. The aperture type—using a fiber whose hollow core acts as an aperture for the light;
2. The apertureless type—that uses a sharp tip. Near-field scanning optical microscopy (NSOM) is the general term for STM, AFM and even SFM-TERS. While ANSOM (Aperture NSOM) is mainly for fiber-type tips or cantilever tips with a hole at the tip end.

Sometimes, it also results that TERS is combined with other methods:

1. TERS can be used in scanning probe microscopy (SPM).
2. TERS can also be coupled to a scanning tunneling microscope (STM-TERS). In such a case, the enhancement will be produced by the gap mode plasmon between the metallic probe and the metallic substrate [27].
3. Raman microscope coupled with atomic force microscope (AFM-TERS), which is widely used in live bio samples [28].
4. Shear force microscopy based TERS system (SFM-TERS).
5. Near-field scanning optical microscopy (NSOM) based TERS system (NSOM-TERS). Raman signals are collected through the same fiber that delivers the excitation light.

TERS has been used for several applications: imaging of single atoms, imaging of internal molecular structure [29,30,31,32], imaging of vibrational normal modes of single porphyrin molecules [33], demonstration of DNA sequencing [34], and ion-selective, atom-resolved imaging of a 2D Cu_2_N insulator using a functionalized tip [35]. Several works looked at the geometry of the tips for specific optical resonance and enhancement purposes [36] and for large opening angles [37].

In this research, the main goal is to optimize the tip nanostructure geometry and material, towards obtaining the optimal density of multiple nanostructures per future array. The final planned pixel will be built with a nanostructured substrate composed of an array of projections or cavities. The shape of these nanostructures and the thickness of their metallic layer (Ag, Au, and Al) can be tuned to deliver the maximal enhancement at the desired wavelength. The number and arrangement of nanostructures was optimized to obtain maximal responsivity.

## 2. Numerical Method: The Finite Element Method (FEM) for PDEs

### 2.1. Best Known Methods (BKM) Choice and Usage

Complementary methods, analytical and numerical, were used in order to accurately model SERS. In this research, the primary numerical approach was the Finite Elements Method (FEM), applied in COMSOL platform tool, and combined with algorithmic optimization algorithms such as Simulated Annealing and Method of Simplexes. Additional simulation programs such as CST, DDSCAT, and MEEPS, based on alternate methods like Method of Moments (MoM), Discrete Dipole Approximation (DDA) and Finite Differences Time Domain (FDTD), respectively, have been considered as necessary, but are not presented in this study. The enhancement of Raman emission from emitters, which are volume-dispersed in a fluid, as well as the possibility of near-field detection through plasmonic antennae, will require the use of simulation approaches, which go beyond the current approach based on surface-integrals in the *E*^4^ approximation. Numerical simulation of propagation of incoherent radiation are performed using a Monte-Carlo approach for individual source phases, as well as a continuum model.

### 2.2. Mesh Shapes and Sizes

Finite Elements Method (FEM) is used in multi-physics software packages in order to support the design and simulation of physical devices and phenomena [38]. The physical equations are discretized on a mesh. The FEM primary advantage is the use of a mesh, which can be variable-sized, with elements of various shapes, making it much better suited to curved geometries. The function of interest *u*(*r*) is expanded in terms of basic functions (or “shape functions”) tailored to the mesh, $\left\{\varphi_{i}\left(\bar{r}\right)\right\}$, $u\left(\bar{r}\right)=\overset{N}{\underset{i=1}{\sum }}u_{i}\varphi_{i}\left(\bar{r}\right)$. The wave equation becomes a system of equation for *u_i_*. A solution is achieved using direct or iterative linear and non-linear solvers. The heart of any nonlinear solver, whether in Matlab, Comsol, or elsewhere is some version of the Newton–Raphson iterations: at every stage, the derivative is used to estimate the distance to the solution. The algorithm continues until the error converges below some minimal value.

### 2.3. Boundary Conditions and Symmetries

The final stage in building a simulation is the choice of boundary conditions. The appropriate choice of boundary conditions implements symmetries which can reduce the domain-size. Mirror symmetries, implemented by reflecting boundary conditions, cut the domain-size in half. Periodic boundary conditions implement a discrete (lattice) translation symmetry, which allows the simulation of an infinite array. They can also be used to implement a discrete rotation symmetry, thereby reducing the domain-size by some finite fraction—a third, a fourth etc. Continuous symmetries, such as axial—i.e., translational—symmetry, and cylindrical—i.e., rotational—symmetry can reduce a three-dimensional simulation to two dimensions. Open or absorbing boundary conditions allow the simulation of infinite domains. “Scattering Boundary Conditions” and “Impedance Boundary Conditions” are well-known varieties; “Port Boundary Conditions” are a proprietary type implemented in Comsol. An alternative method makes use of a region of highly dissipative propagation known as a “Perfectly Matched Layer” (PML). A similar method, known as an Infinite Domain, makes use of a non-linear spatial transformation. Very thin layers—regions of high aspect ratio—are a computational obstacle insofar as they require very fine meshes. This can be avoided, and good accuracy can be obtained by replacing the region with boundary conditions which relate the fields on either side by extrapolation. This is computed based on the material characteristic of the layer—the electrical resistance, thermal conductance, optical transmittance, etc. [32].

## 3. Analytical Method: Models and Properties of Metallic Nano-Particles

### 3.1. Analytical Method: Models and Properties of Metallic Nano-Particles

An electromagnetic source excites the nanostructure, and by observing the absorption and scattering cross-section, its electromagnetic properties can be determined. When an impinging electromagnetic wave with an appropriate incident wavelength illuminates a metallic nano-particle, the metals’ free electrons start oscillating collectively. Such oscillations lead to the propagation of strong surface waves [39,40], also known as Propagating Surface Plasmon Polaritons (PSPP). The resonant optical properties of nano-particles can be studied, starting with their polarizability expressions. The polarizability value strongly depends on the nano-particle geometry, particularly on its size, shape, inclusion composition, and the surrounding dielectric environment refractive index.

### 3.2. Electrostatic Approximation and Mie Theory for Metallic Sphere

The solution to the electrostatic problem for a sphere is well known [41]. The electric field solution inside the sphere is given by:

(1) $$E_{In}=\frac{3\varepsilon_{e}}{\varepsilon_{i}+2\varepsilon_{e}}E_{inc}$$

where $E_{In}$ is the electric field inside the sphere nano-particle,$\;E_{inc}$ is the incident electric field, $\varepsilon_{e}$ is the surrounding dielectric environment permittivity, $\varepsilon_{i}\;$is the inclusion dielectric permittivity. Dimensionless polarizability for the electrostatic problem for a sphere is given by:

(2) $$\beta_{s}=\frac{\varepsilon_{i}-\varepsilon_{e}}{\varepsilon_{i}+2\varepsilon_{e}}$$

The Electro-Static Approximation is very limited because it does not consider size-related effects, therefore the energy conservation is only approximated. Depolarization and radiative corrections to the Electro-Static Approximation (ESA) approach have been made through using Mie theory [1]:

(3) $$\beta_{s}^{Mie}=\frac{\beta_{s}}{1-{\left(K_{e}a\right)}^{2}\left[1-\frac{2\varepsilon_{i}+1}{5\left(\varepsilon_{i}-1\right)}\right]\beta_{s}-\frac{2}{3}i{\left(K_{e}a\right)}^{2}\beta_{s}}$$

where:

- $\beta_{s}^{Mie}$ is the corrected polarization by Mie theory;
- $\beta_{s}$ is the ESA approximation polarization;
- $K_{e}=2\pi n/\lambda$ is the wavenumber in the medium;
- a is the size of the radius of the sphere.

Far field properties such as absorption and scattering cross-section can calculated by using the polarizability $\beta_{s}^{Mie}$:

(4) $$\sigma_{ext}=4\pi K_{e}a^{3}Im\left(\beta_{s}^{Mie}\right)$$

(5) $$\sigma_{sca}=\frac{8\pi a^{2}}{3}{\left(K_{e}a\right)}^{4}{\left|\beta_{s}^{Mie}\right|}^{2}$$

The extinction cross-section is the sum of the scattering and absorption cross-section. Therefore, the absorption cross-section is:

(6) $$\sigma_{abs}=\sigma_{ext}-\sigma_{sca}$$

Scattering and absorption cross-section provides great insight for the study of electromagnetic properties of a nano-particle. However, the following assumptions must be made:

- The resonant behavior of the individual nano-particle can be studied in terms of quasi-static approximation; therefore, the size of a nano-particle must be smaller than the wavelength of the light source [42]. The electromagnetic field is approximately constant over the particle volume for small nano-particles.
- The nano-particle macroscopic electromagnetic behavior can be related to its polarizability only if the considered particle is homogeneous, and the surrounding material is a homogeneous, isotropic, and non-absorbing medium.

### 3.3. Analytical Models of Prolate Spheroid Nano-Particles

The analytical models of prolate spheroid nano-particles, which link the electromagnetic nano-particle properties to their geometrical and structural parameters, are presented in order to describe their resonant behavior. Considering the electric field $=E_{0}\hat{z}=-\nabla \varphi_{0}$, the solution for ESA problem of prolate spheroid:

(7) $$\varphi_{in}=\frac{\varphi_{0}}{1+L_{3}\frac{\varepsilon_{i}-\varepsilon_{e}}{\varepsilon_{e}}}=\frac{3\varepsilon_{e}}{3L_{3}\varepsilon_{i}+\varepsilon_{e}\left(3-3L_{3}\right)}\varphi_{0}$$

As was discussed for the sphere nano-particle, the dimensionless polarizability for excitation along the Z axis is:

(8) $$\beta_{s}=\frac{\varepsilon_{i}-\varepsilon_{e}}{3L_{3}\varepsilon_{i}+\varepsilon_{e}\left(3-3L_{3}\right)}$$

where $L_{3}$ is the depolarization factor and is calculated by the following equation:

(9) $$L_{3}=\frac{1}{2e^{2}}\left[1-\frac{1-e^{2}}{2e}\ln\left(\frac{1+e}{1-e}\right)\right]$$

(10) $$e=\sqrt{1-{\left(\frac{b}{a}\right)}^{2}}$$

**Figure 1:**
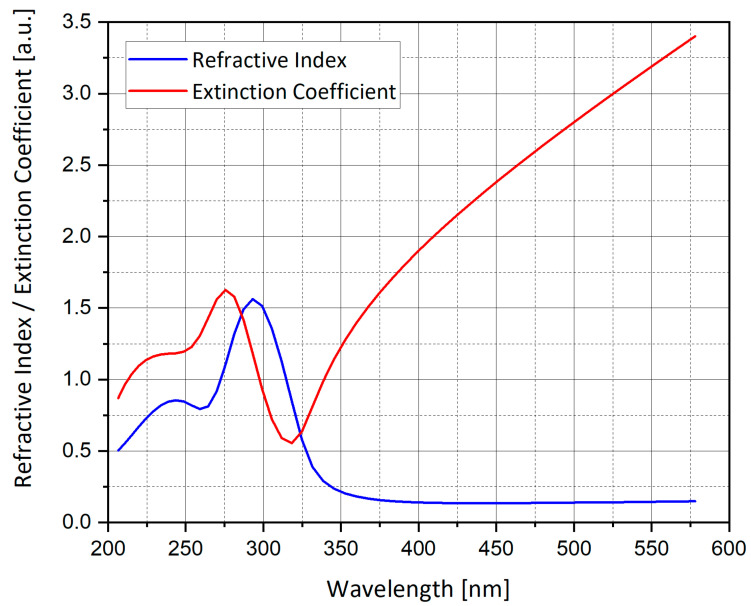
Refractive index and extinction coefficient of silver (Ag).

**Figure 2:**
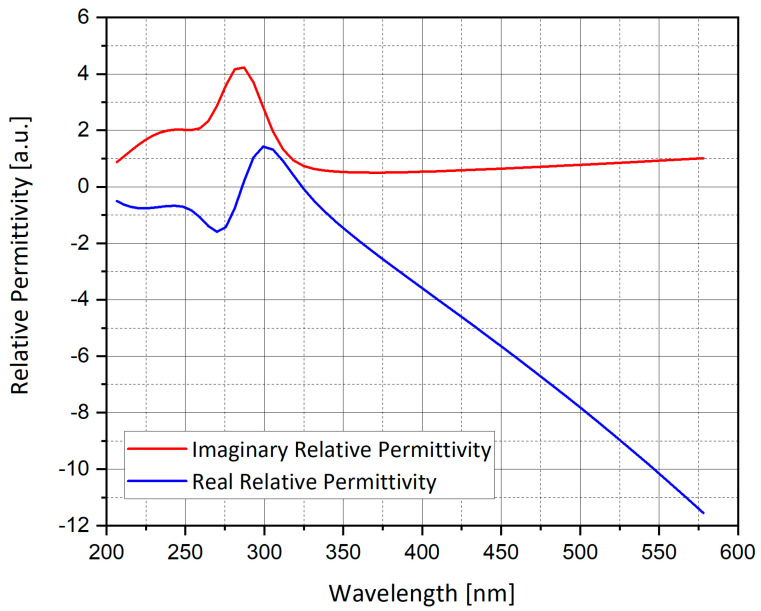
Real and imaginary part of the relative permittivity of silver (Ag).

**Figure 3:**
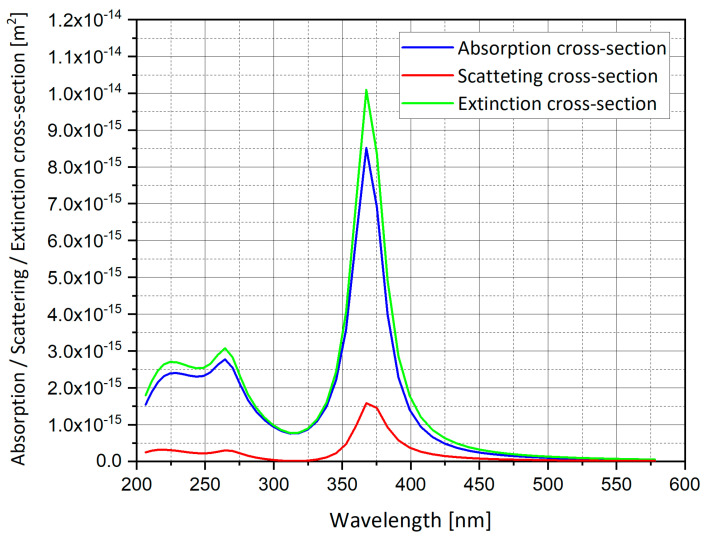
Absorption, scattering and extinction cross-section of silver (Ag) spheroid nano-particle.

The $L_{3}$ factor of a nano-particle plays a crucial role in the polarizability’s resonant behavior for the enhancement of localized surface plasmon resonance (LSPR) strength. The far field properties are obtained by using the dipolar approximation. The extinction cross-section equation:

(11) $$\sigma_{ext}=4\pi K_{e}abcIm\left(\beta_{s}\right)$$

(12) $$\sigma_{sca}=\frac{8\pi {\left(abc\right)}^{2}}{3}{\left(K_{e}\right)}^{4}{\left|\beta_{s}\right|}^{2}$$

where *a*, *b* and *c* are the spheroid axes, and the absorption cross-section is the same as Equation (6). A Matlab code was used to plot the graphs of the extinction cross-section for silver sphere nano-particle (L ≈ 1/3), as presented in Figure 1, Figure 2 and Figure 3.

Figure 1, Figure 2 and Figure 3 show the analytical model for silver nano-particle extinction cross-section. Absorption cross-section is higher than scattering cross-section for a small nano-particle, because as a particle grows it becomes closer to the size of the light wavelength, therefore scattering interaction occurs more often compared to a small particle which absorbs the photon and dissipate the photon energy as heat. Absorption and scattering cross-section are competing phenomena and have different application and measurement techniques.

**Figure 4:**
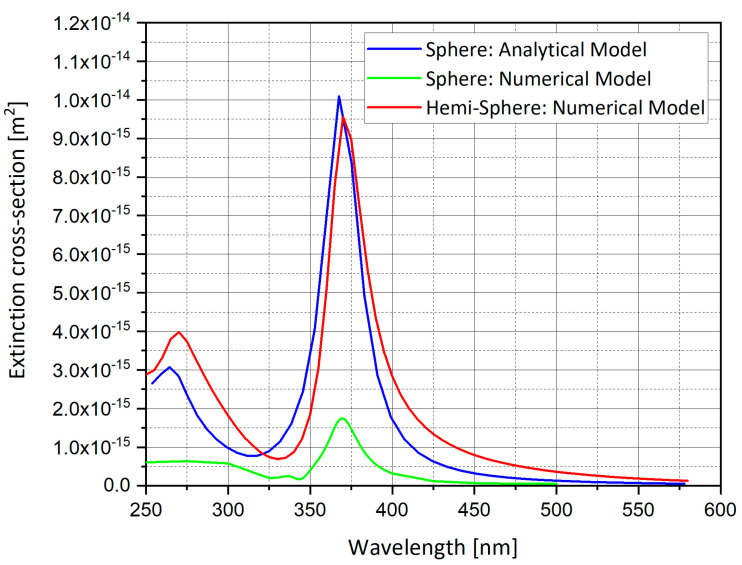
Analytical and numerical model comparison for extinction cross-section of silver (Ag) sphere and hemi-sphere.

Figure 4 compares the analytical model with the numerical model of a silver sphere nano-particle and shows the difference between sphere geometry and hemi-sphere geometry. The analytical and numerical models matched almost completely. Moreover, the hemi-sphere geometry follows the same pattern as the sphere geometry but the values of the extinction cross-section for the hemi-sphere are smaller.

**Figure 5:**
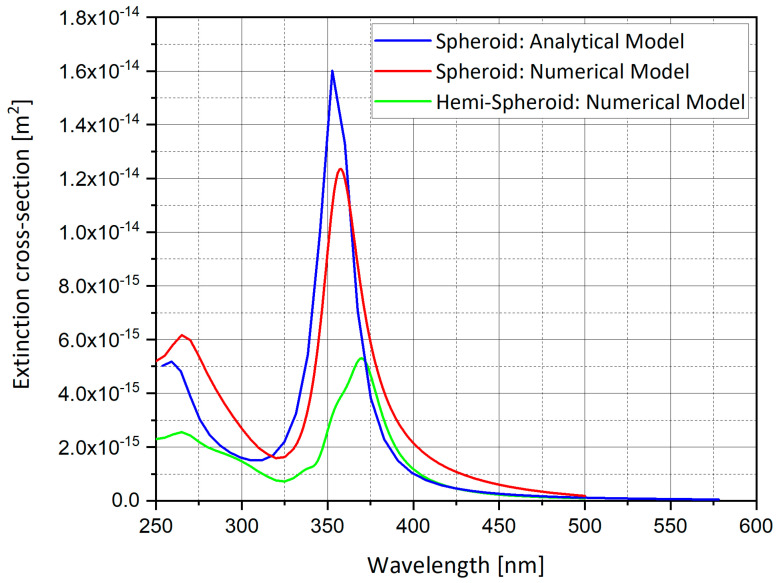
Analytical and numerical model comparison for extinction cross-section of silver (Ag) spheroid and hemi-spheroid.

Figure 5 compares the analytical model with the numerical model of the silver spheroid nano-particle and shows the difference between spheroid geometry and hemi-spheroid geometry. The analytical and numerical models matched almost completely, moreover the hemi-spheroid geometry follows the pattern as the sphere geometry but the extinction cross-section values for the hemi-spheroid are smaller and the peak is slightly off.

## 4. Simulation Results

### 4.1. Field Enhancement Factor (FFF)

**Figure 6:**
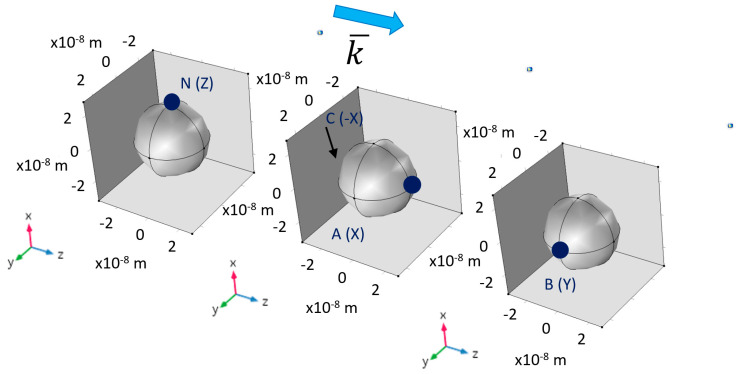
Illustration example of Comsol simulation of the points (N), (A), and (B). The field enhancement is displayed at the points (N), (A), and (B) and some of their polar pairs of the nano-spheres.

The field enhancement factor for a silver sphere (Figure 6) was calculated by numerical simulation to validate the method of simulation. When comparing the enhancement obtained by a nanostructure and a nano-cavity of the same shape, a curious duality is noted between particles and cavities which exchanges the roles of prolate and oblate spheroids, and between the major and minor axes of any particular spheroid; it is significant in choosing the optimal shape for a given excitation polarization and vice versa. Stratified nanostructures—nano-shells—introduce some freedom in tuning the resonant frequency; the predictions of the Electro-static Approximation (ESA) will be compared to the propagating simulation for the case of spheres. A simplified model of the actual device was studied, by simulating a system of two structures and studying their mutual influence as a function of separation. Following this, a pixel design based on a finite array of nanostructures was studied.

### 4.2. Parameters, Operators and Variables

System constants are necessary for constructing the CAD geometry and values needed in parametric sweeps were defined as parameters (depicted in Table 1). Values that needed to be changed throughout the simulation were defined as variables in a way that made realizing the complex expression more manageable, Table 2.

### 4.3. Geometric Structures, Physics Definitions, Materials and Mesh

**Figure 7:**
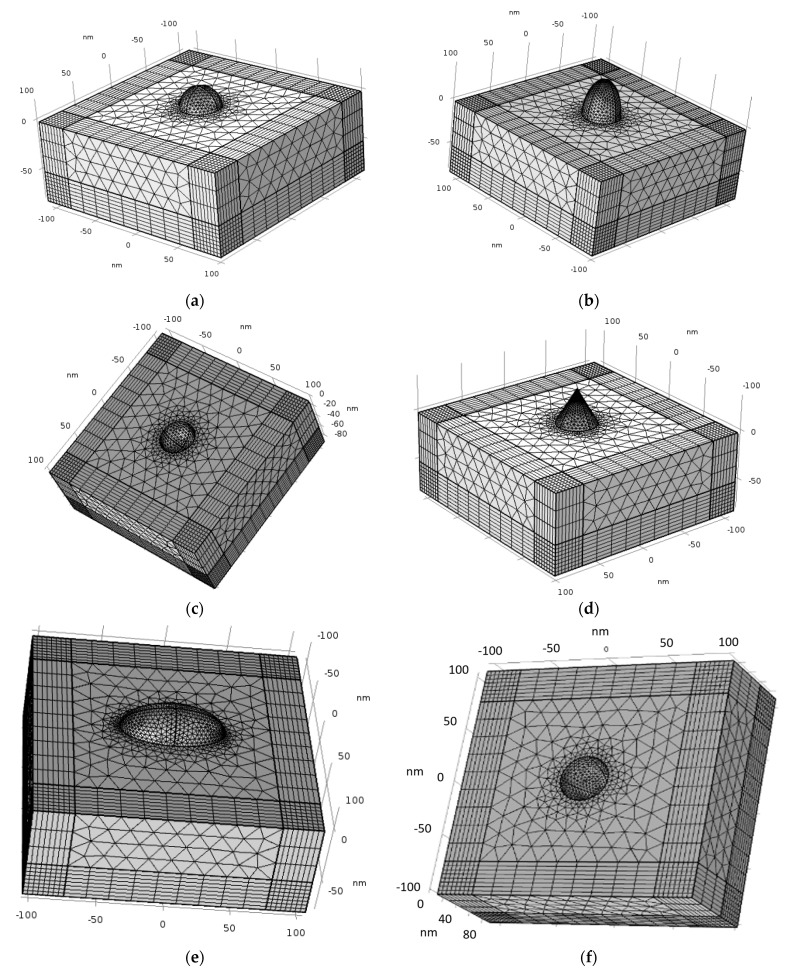

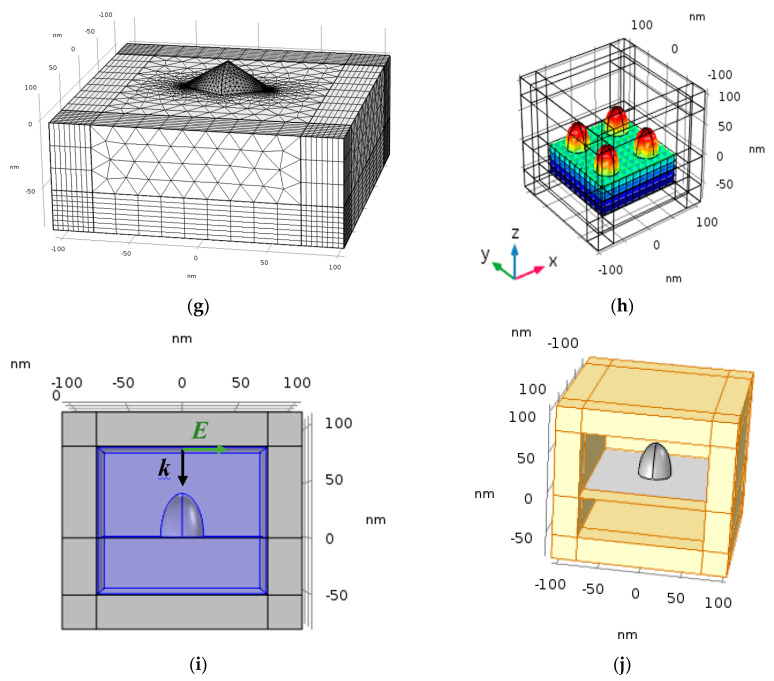
Tip structure different geometries: (**a**) hemi-sphere nano-particle; (**b**) hemi-spheroid nano-particle; (**c**) cavity; (**d**) nano-cone; (**e**) ellipsoidal rod; (**f**) ellipsoidal cavity; (**g**) double nano-cone; (**h**) SERS/TERS square; (**i**) the physical domain and the port input electric field excitation; (**j**) the PML domain.

The geometric structures checked in the simulations are described in the following figures: hemi-sphere (Figure 7a), hemi-spheroid (Figure 7b), cavity (Figure 7c), nano-cone (Figure 7d), ellipsoidal rod (Figure 7e), ellipsoidal cavity (Figure 7f), double nano-cone (Figure 7g). Multi-tips arrays are also presented in Figure 7h. The Wave Optics module was used in all simulations by using the electromagnetic waves frequency domain (ewfd) model. In the first step of the simulation, the full field was simulated in the physical domain as shown in Figure 7i, and in the second step the scattered field was simulated in all domains. A periodic boundary condition was used, and PML as shown in Figure 7j. The input electric field excitation enters from the top (Figure 7i). The materials used in the simulations, are silver (Ag rakic model) for substrate and nano-particle (Figure 7a), and air for all other domains.

### 4.4. Solvers and Studies

In order to identify the optimal shape and geometry, seven shape studies were performed: hemi-sphere, cavity, hemi-spheroid, nano-cone, ellipsoidal cavity, ellipsoidal rod and double nano-cone. The simulations consist of two steps: simulation of the full field (ewfd) and simulation of the scattered field (ewfd2). All the simulations used an input electrical field with a wavelength in the range of 250–500 nm. While the width of physical geometry W = 150 nm and a parametric sweep for θ = 0, π/6, π/4, and π/3 were, respectively, used in the simulations of the hemi-sphere, cavity, hemi-spheroid, and ellipsoidal cavity (with the exception of θ = π/4). Following are the results.

### 4.5. Hemi-Sphere Geometry Results

**Figure 8:**
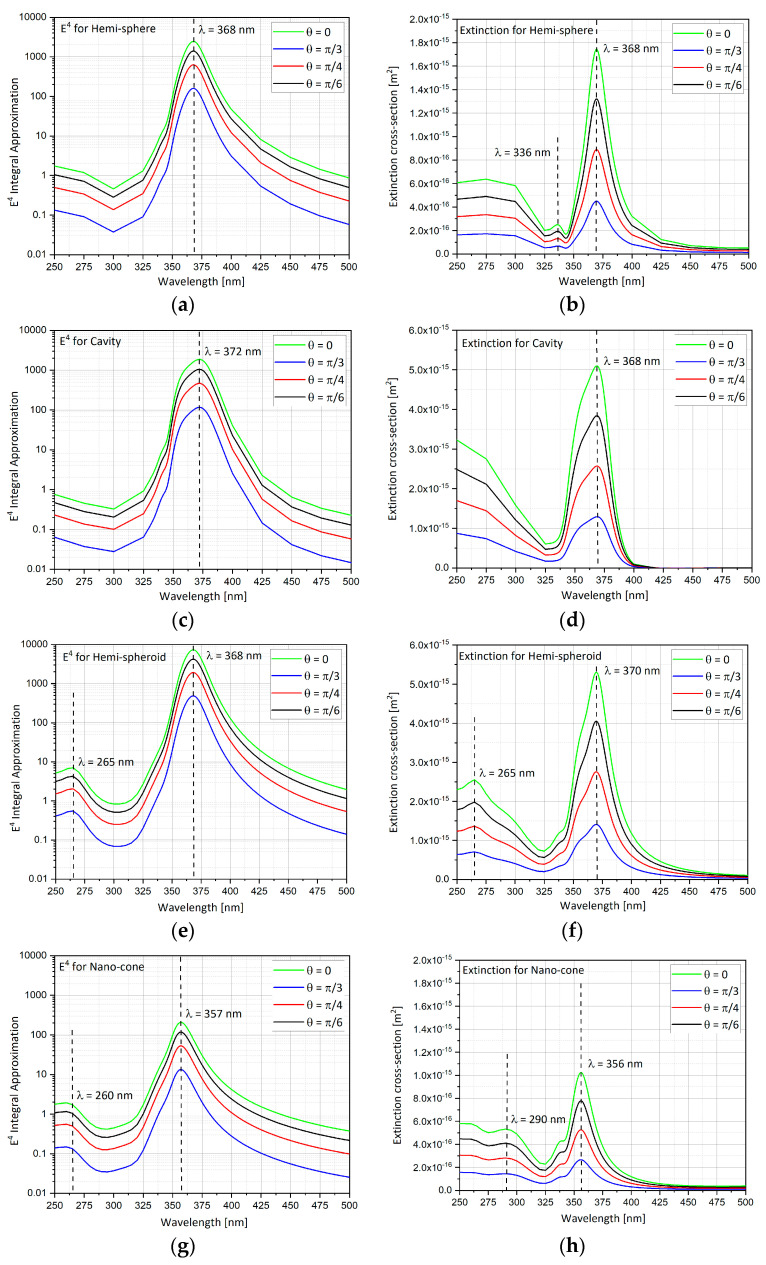
*E*^4^ approximation and extinction cross-section for changing polar angle θ in wavelengths range of λ = 250–500 nm. (**a**) *E*^4^ approximation for hemi-sphere; (**b**) extinction for hemi-sphere; (**c**) *E*^4^ approximation for cavity; (**d**) extinction for cavity; (**e**) *E*^4^ approximation for hemi-spheroid; (**f**) extinction for hemi-spheroid; (**g**) *E*^4^ approximation for nano-cone; (**h**) extinction for nano-cone.

By impinging light in the Z axis, with electric field polarized in the Y axis, with different polar angles θ = 0, π/6, π/4, and π/3 and in different wavelengths, the *E*^4^ approximation and the extinction cross-section can be calculated as, respectively, shown in Figure 8a,b. As the polar angle θ gets bigger, the enhancement factor gets smaller. The peak enhancement is at 368 nm. When the K vector of the input electric field is normal to the substrate with the nano-particle the field enhancement is largest. The extinction cross-section shows the same behavior when changing the polar angle θ as it was in the *E*^4^ calculation above. The peak is at 368 nm, and at θ = 0 is the largest extinction cross-section.

### 4.6. Cavity Geometry Results

As discussed above in the hemi-sphere shape analysis, the *E*^4^ approximation and the extinction cross-section can be calculated (Figure 8c,d). Again, as the polar angle θ gets bigger, the enhancement factor gets smaller. The peak enhancement is at 372 nm. When the K vector of the input electric field is normal to the substrate with the cavity the field enhancement is largest. The extinction cross-section shows the same behavior when changing the polar angle θ as it was in the *E*^4^ calculation above. The peak is at 371 nm, and at θ = 0 the extinction cross-section is the largest.

### 4.7. Hemi-Spheroid Geometry Results

*E*^4^ approximation and extinction cross-section are calculated (Figure 8e,f). The hemi-spheroid that was used here is with eccentricity of 0.866. As polar angle θ gets bigger, the enhancement factor gets smaller. The peak enhancement is at 368 nm. When the K vector of the input electric field is normal to the substrate with the nano-particle, the field enhancement is largest. The extinction cross-section shows the same behavior when changing the polar angle θ as it was in the *E*^4^ calculation above. The peak is at 368 nm, and at θ = 0 the extinction cross-section is the largest.

### 4.8. Nano-Cone Geometry Results

The *E*^4^ approximation and the Extinction cross-section are presented (Figure 8g,h). The nano-cone surface area is the same as the hemi-sphere surface area. As polar angle θ gets bigger, the enhancement factor gets smaller. The peak enhancement is at 357 nm. When the K vector of the input electric field is normal to the substrate with the nano-cone, the field enhancement is largest. The extinction cross-section shows the same behavior when changing the polar angle θ as it was in the *E*^4^ calculation above. The peak is at 355nm, and at θ = 0 the extinction cross-section is the largest.

### 4.9. Ellipsoidal Cavity Geometry Results

**Figure 9:**
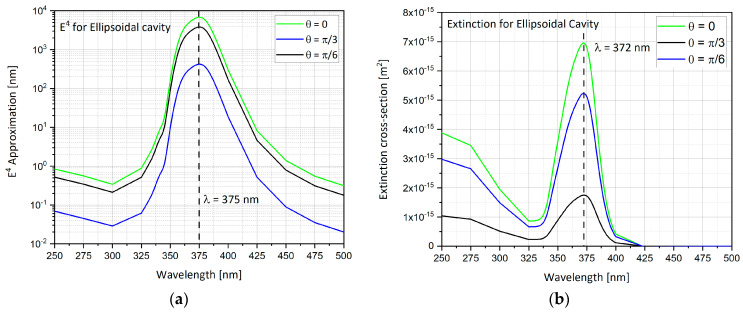
Ellipsoidal cavity results while changing the polar angle θ in a wavelengths range of λ = 250–500 nm. (**a**) *E*^4^ approximation; (**b**) extinction cross-section.

In addition to above standard shapes, additional complex configurations were also analyzed, like the ellipsoidal cavity geometry, analyzed here. As discussed in the hemi-sphere, the *E*^4^ approximation and the extinction cross-section can be calculated (Figure 9a,b). As polar angle θ gets bigger, the enhancement factor gets smaller. The peak enhancement is at 375 nm. When the K vector of the input electric field is normal to the substrate with the cavity, the field enhancement is largest. The extinction cross-section shows the same behavior when changing the polar angle θ as it was in the *E*^4^ calculation above. The peak is at 372 nm, and at θ = 0 the extinction cross-section is the largest.

### 4.10. Ellipsoidal Rod Geometry Results

**Figure 10:**
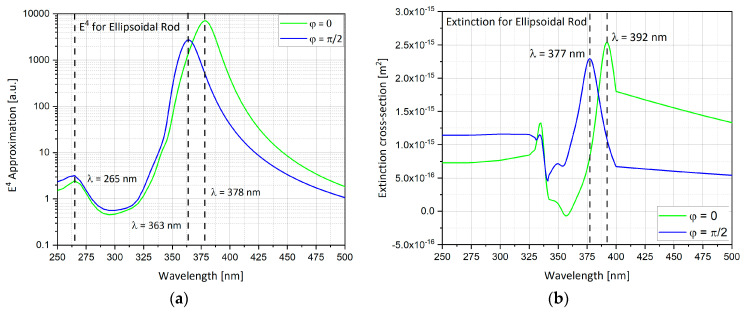
Ellipsoidal rod results while changing the polar angle θ in a wavelengths range of λ = 250–500 nm. (**a**) *E*^4^ approximation; (**b**) extinction cross-section.

The ellipsoidal rod geometry is analyzed in this section. As discussed in the hemi-sphere the *E*^4^ approximation and the extinction cross-section can be calculated (Figure 10). The ellipsoidal rod that was used here is with eccentricity of 0.866 in the Y direction. Contrary to other geometries, this geometry is sensitive to the electric field polarization. When the electric field polarization is in the Y direction (*φ* = 0), localized surface plasmon (LSP) is produced like a dipole in accordance with the electric field polarization. This time, the peak enhancement is at 378 nm. The same happens in the perpendicular direction X for polarized electric field in the X direction (*φ* = π/2). The peak enhancement is at 363 nm. When the electric field polarization is *φ* = 0 the peak that is produced is higher than the peak produced by φ = π/2.

### 4.11. Double Nano-Cone Geometry Results

**Figure 11:**
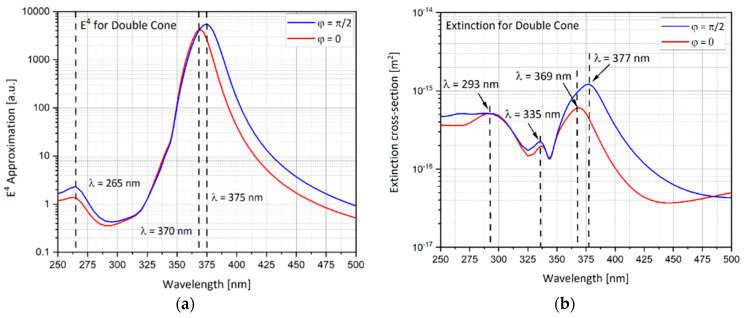
Double nano-cone results while changing the polar angle θ in a wavelengths range of λ = 250–500 nm. (**a**) *E*^4^ approximation; (**b**) extinction cross-section.

The double nano-cone geometry is analyzed in this section. As discussed in the hemi-sphere, the $E^{4}$ approximation and the extinction cross-section can be calculated (Figure 11). As discussed in the ellipsoidal rod, this geometry is sensitive to the electric field polarization. When the electric field polarization is in the Y direction (φ = 0), localized surface plasmon (LSP) is produced like a dipole in accordance with the electric field polarization. The peak enhancement for polarization in Y direction (φ=0) is at 370 nm. The same happens in the perpendicular direction X for the polarized electric field in the X direction (φ = π/2). The peak enhancement for polarization in Y direction (φ=π/2) is at 375 nm. When the electric field polarization is φ=π/2, the produced peak is higher than the peak produced by φ=0.

### 4.12. Results Comparison between Different Nano-Particles Geometries

**Figure 12:**
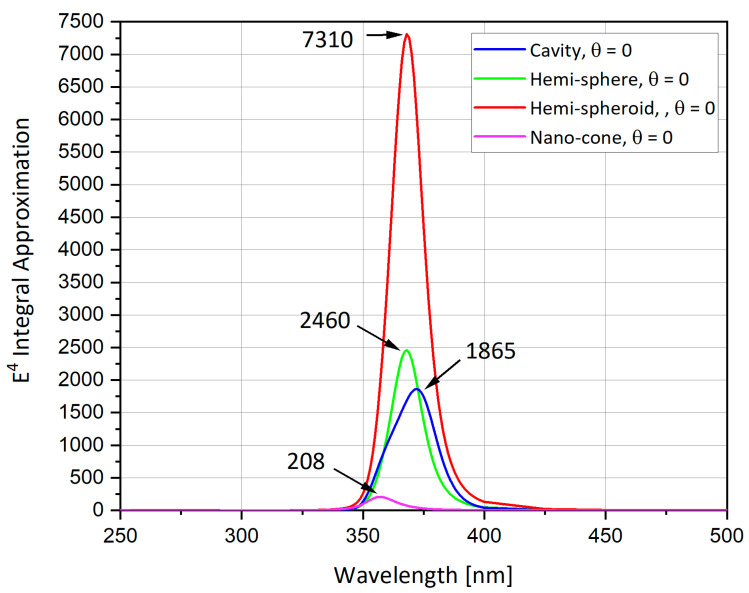
Surface-Enhanced Raman Scattering (SERS) EF: comparison between different nano-particle geometries.

**Figure 13:**
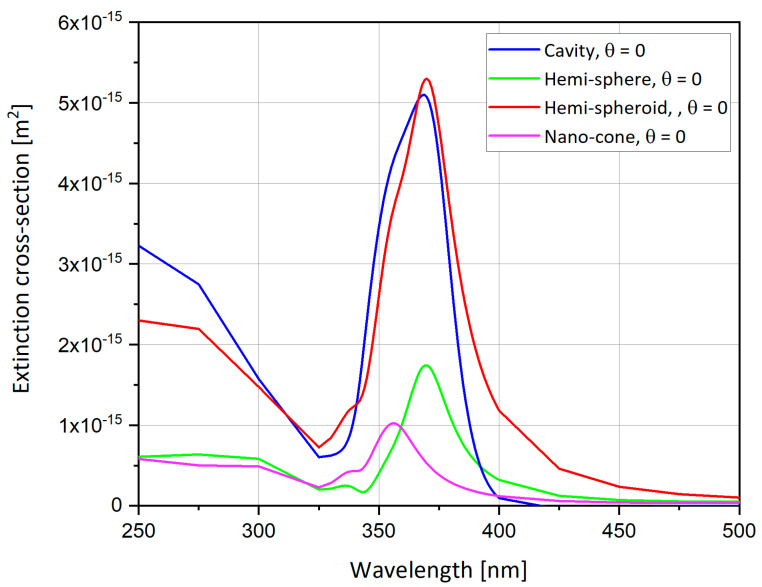
Extinction cross-section: comparison between different nano-particle geometries.

The hemi-sphere, cavity, hemi-spheroid and the nano-cone shapes are now compared. As shown in Figure 12, the spheroid has the largest SERS Enhancement Factor (EF) reaching up to 7300, followed by the sphere with SERS EF of 2500, the cavity with SERS EF around 1800 and finally the nano-cone with SERS EF less than 300. Figure 13 shows the comparison of the extinction cross-section between geometries and the spheroidal cavity and the hemi-spheroid shows the highest extinction cross-section values, but the extinction cross-section is highest in the ellipsoidal cavity by far reaching up to 7 · 10^-15^ m^2^, as shown from Figure 9b. In order to summarize the seven presented options and to classify them by preference criteria, Table 3 includes the main parameters and obtained values.

### 4.13. Silver vs. Gold vs. Aluminum

**Figure 14:**
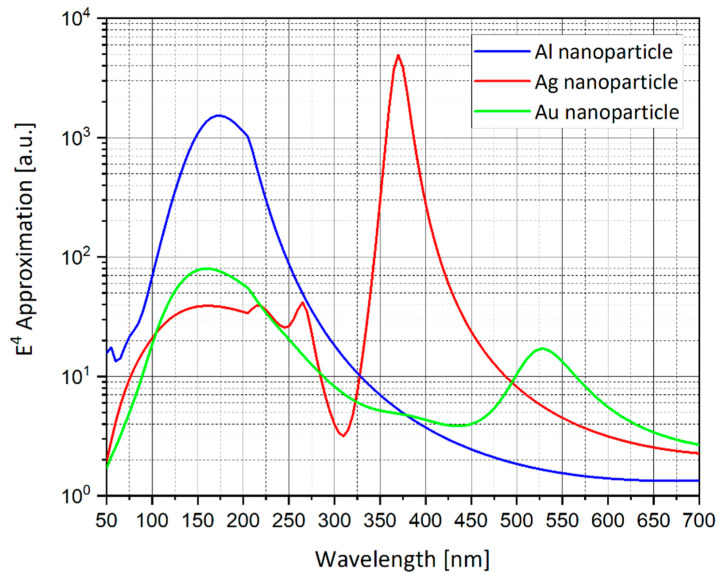
Field Enhancement vs. wavelength for an incident plane wave on a silver (Ag), gold (Au) and aluminum (Al) 20 nm-radius sphere. The *E*^4^ approximation for the electric field enhancement is displayed. Relevant wavelength peaks: silver at λ = 370 nm, gold at λ = 530 nm, aluminum at λ = 175 nm.

**Figure 15:**
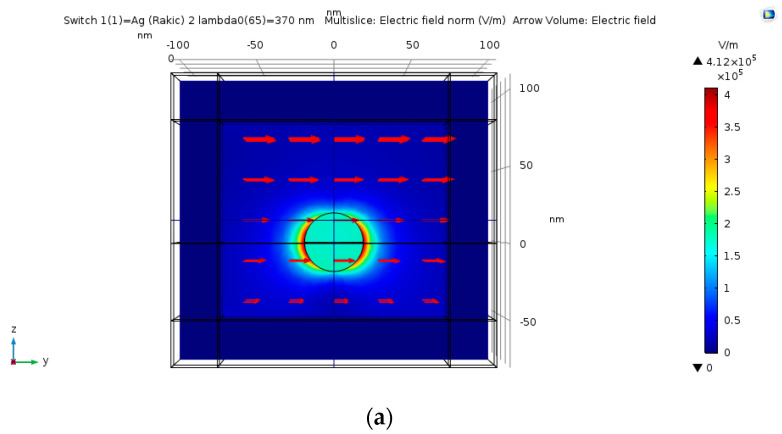

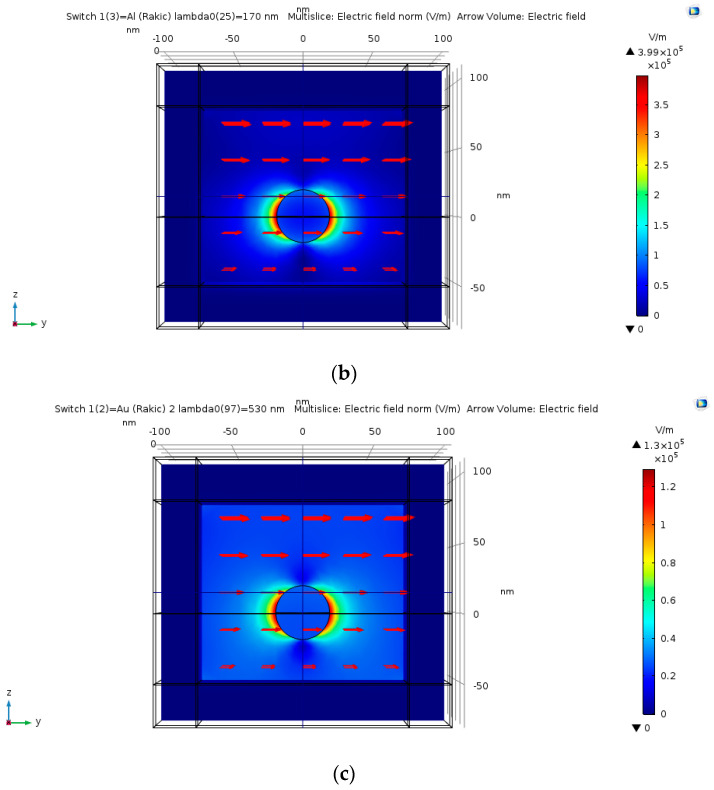
Simulations of silver (**a**), aluminum (**b**) and gold (**c**) nano-particle with the input of electric field in the Y direction and with k vector in the -Z direction.

As part of the optimization process, the identification of the tip material was also investigated. The simulations compare between silver (Ag), gold (Au) and aluminum (Al). The goal was to determine which material is more suited for higher SERS EF. As shown in Figure 14 and Figure 15, silver/gold/aluminum nano-sphere with radius of 20 nm is simulated. The silver nano-sphere produces the highest peak of electric field enhancement, then the aluminum nano-sphere and lastly the gold nano-sphere, but the peaks are shown to be at different wavelengths. Silver seems to be more suitable for field enhancement in the region of wavelengths of 325–495 nm as shown in Figure 7.

### 4.14. Nano-Shells Tuning

**Figure 16:**
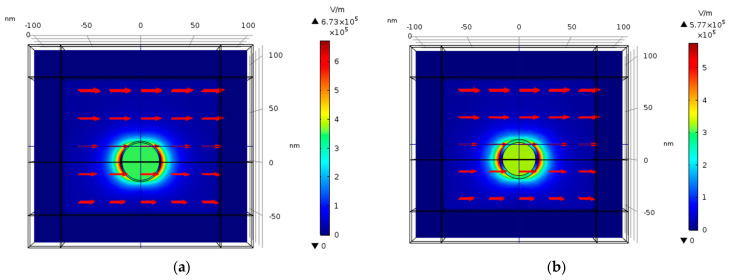

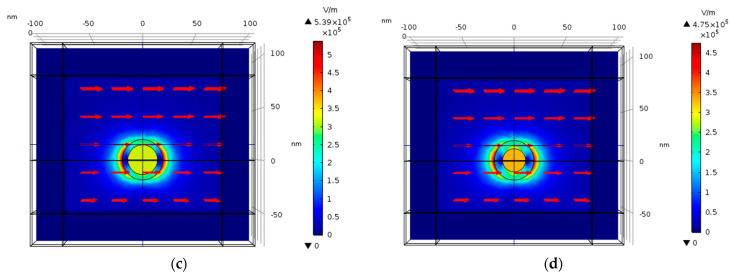
Simulation of nano-shells with thickness (T) ranging from 1.433 to 8.303 nm. The impinging electric field is polarized in the Y direction as shown by the red arrows and the wave front direction (K vector) is in the -Z direction. Δ = 0.2 (**a**), Δ = 0.4 (**b**), Δ = 0.6 (**c**), and Δ = 0.8 (**d**), where Δ = 1-(r/R)^3^.

**Figure 17:**
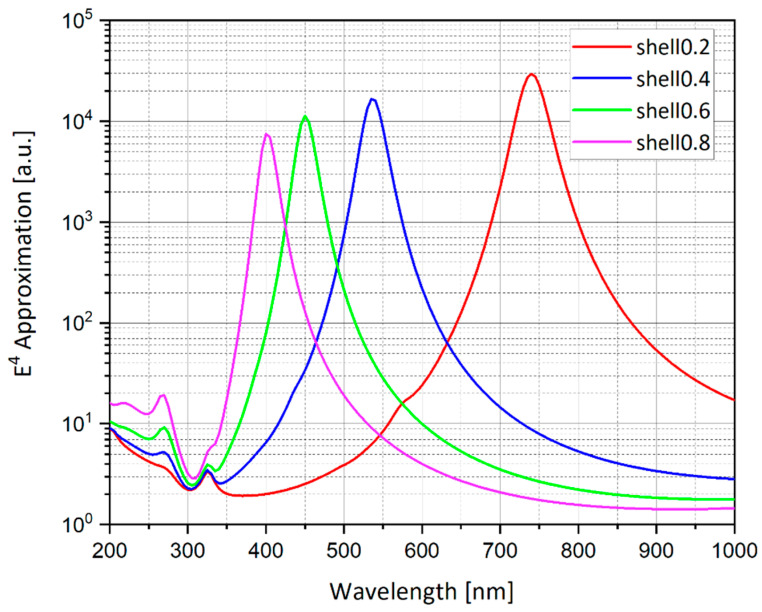
Enhancement curves for spherical nano-shells with outer radius (R) of 20 nm and inner radius (r) such that the nano-shell thickness is T = R − r. The nano-shells thickness ranges between T(Δ = 0.2) = 1.433 nm and T(Δ = 0.8) = 8.303 nm. The shells are excited by a plane wave. The resonance peak shifts depending on the shell thickness. Moreover, the electric field enhancement gets bigger as the nano-shell thickness decreases.

Stratified nanostructures—nano-shells, first introduced by (Halas, 2005) [43]—are seen to introduce some freedom in tuning the resonant frequency (Figure 16 and Figure 17). A silver nano-shell with an outer radius (R) of 20 nm and inner radius (r) 11.697–18.567 nm is simulated.

As shown in Figure 17, the nano-shell thickness T = R − r provides a freedom in tuning the resonant wavelength. Moreover, as the shell thickness becomes smaller, the electric field enhancement grows. The sphere has external radius R, internal radius r, and hence thickness R − r. In the ESA, the field enhancement at the North Pole (N) is:

(13) $$M={\left|\frac{\epsilon \epsilon_{M}+\frac{2}{3}\left(\epsilon -\epsilon_{M}\right)\epsilon \Delta }{\epsilon \epsilon_{M}+2{\left(\frac{\epsilon -\epsilon_{M}}{3}\right)}^{2}\Delta }\right|}^{2}$$

where:

(14) $$\Delta \;=\left(1-\frac{r^{3}}{R^{3}}\right)$$

**Figure 18:**
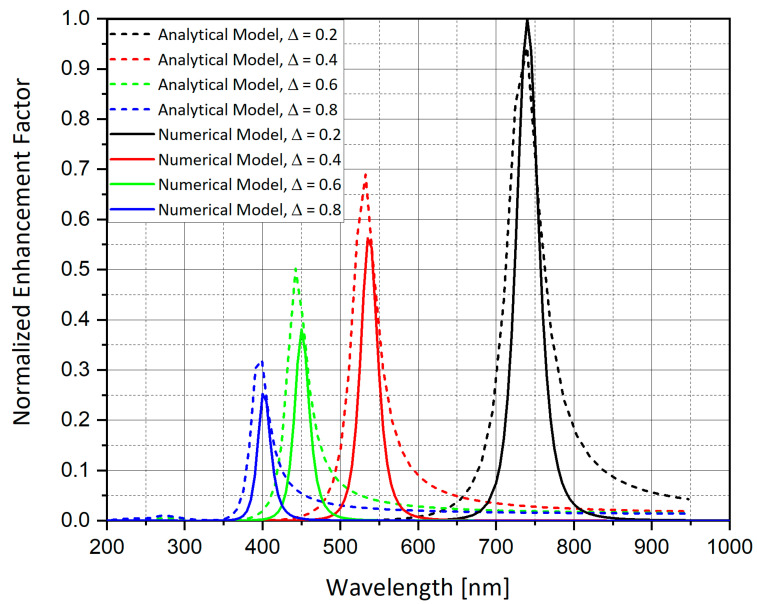
Analytical and numerical models for the normalized enhancement factor.

The solid sphere corresponds to Δ = 1 while the shell of vanishing thickness is described by Δ = 0. In the latter case *M*→1, as consistency demands. Resonance occurs at when (13) is maximal. For a solid structure, the resonance is achieved for a particular wavelength, determined by the form of ϵ(λ). By contrast, expression (13) can be maximized for any value of λ by setting appropriate Δ. Thus, one may choose a convenient wavelength and achieve resonance by tuning the thickness of the shell. This analytical model is compared to the *E*^4^ approximation calculation in the numerical simulation of nano-shells as shown in Figure 18. In the simulation, Δ = 0.2, 0.4, 0.6 and 0.8, respectively, as shown in Figure 16 and Figure 17.

As shown in Figure 18, the analytical and numerical calculations for the normalized enhancement factor are matched on the resonance wavelength.

### 4.15. Multiple Nanostructures Mutual Influence

#### 4.15.1. The Influence of Separation between Nanostructures

**Figure 19:**
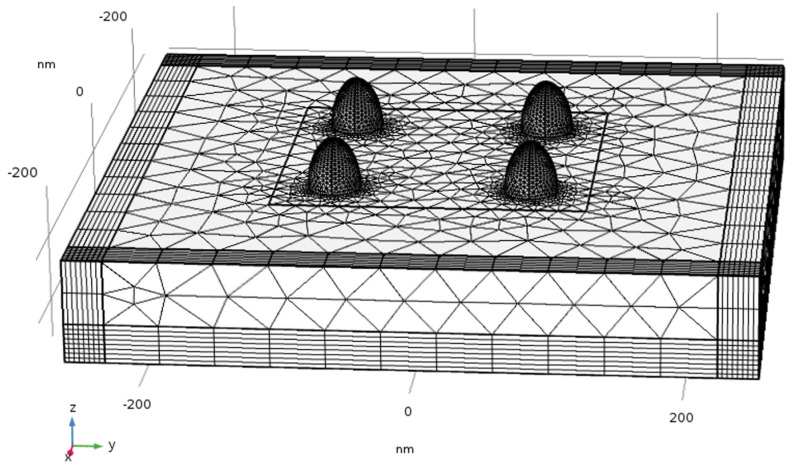
Simulation configuration of neighboring silver hemi-spheroid.

**Figure 20:**
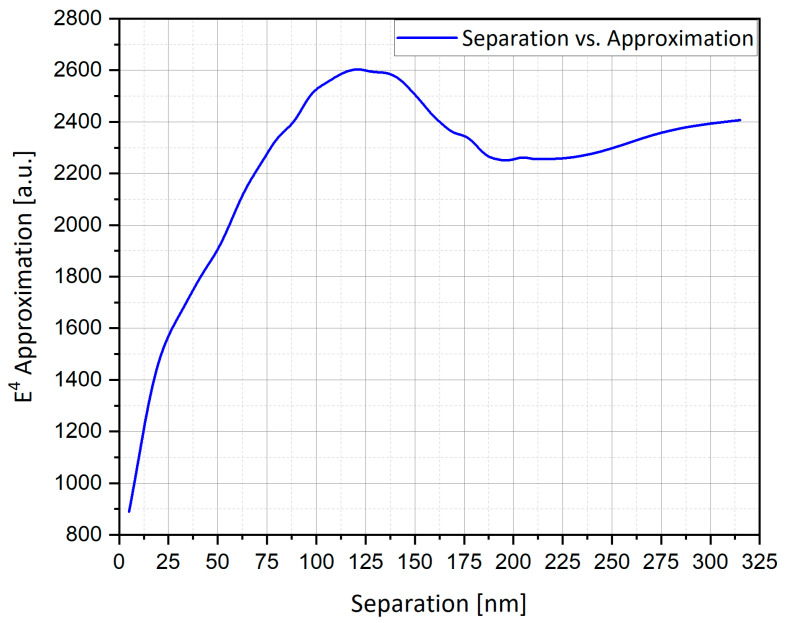
Enhancement vs. separation, for four silver hemi-spheroid in a plane wave, polarized orthogonally to axis of separation. Suppression is observed at short distances. At ~110 nm, the radiation field dominates the localized surface plasmon (LSP) (near) field.

A simplified model of the actual device was studied by simulating a system of four structures in a box with the width of physical geometry W = 450 nm (Figure 19) and studying their mutual influence as a function of separation (Figure 20). This is the $E^{4}$ approximation vs. the separation.

**Figure 21:**
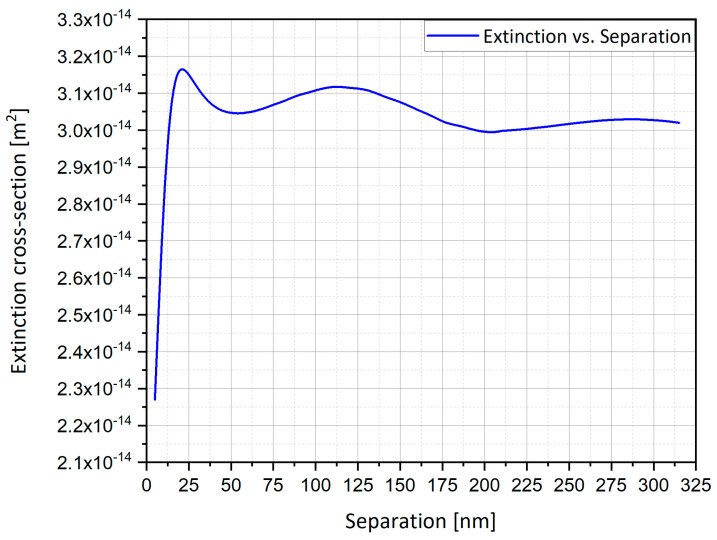
Extinction cross-section vs. separation for four silver spheres in an oscillating electric field in the Y direction.

The mutual influence of neighboring nanostructures was investigated numerically, by following the total integrated enhancement as a function of the separation. Figure 21 displays the extinction cross-section from four particles of silver (Ag) hemi-spheroid subject to oscillating electric field; one clearly discerns that the graph is leveled for large distances and starts decreasing at smaller distances, beneath ~100 nm (there is no maximum because the number of structures is constant). The mutual influence is thus negligible at micrometric distances—the order of a pixel—while at nanometric separation it becomes significant.

**Figure 22:**
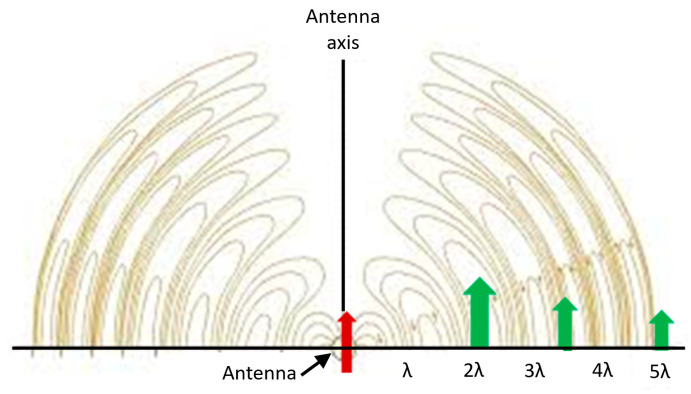
The radiation pattern of a dipole oscillator. The field on the axis is parallel to the dipole leading to enhancement.

The appearance of a maximum (in Figure 20) at ~110 nm may seem surprising. However, the expectations outlined above were based on the ESA. In practice, the field of a dynamic radiating sphere is like that of a dipole (Figure 22). It includes several terms, particularly the local field—this is the LSP field and is the only term seen in the ESA, i.e., by a uniform field. This term causes suppression. It varies ~1/r^3^, the inverse cube of the separation. It also includes the radiation field—this term is only excited by an oscillating field. On the equator, the radiation field is parallel to the source dipole; hence, it causes mutual enhancement, as illustrated in Figure 20 and Figure 22. This term decays as 1/r. Competing phenomena of mutual dipole suppression and enhancement and—at distances smaller than ~5 nm—of gap or hybrid plasmons [1], lead to an optimal separation for maximal total enhancement.

#### 4.15.2. The Comparison between Preliminary Results for the Optimal Separation

Preliminary results, as shown in Figure 20 and Figure 21, present two different optimal separations of nano-particles:

- At a separation of ~110 nm (Figure 20), the highest field enhancement is produced.
- At a separation of ~20 nm (Figure 21), the highest extinction cross-section is produced.

**Figure 23:**
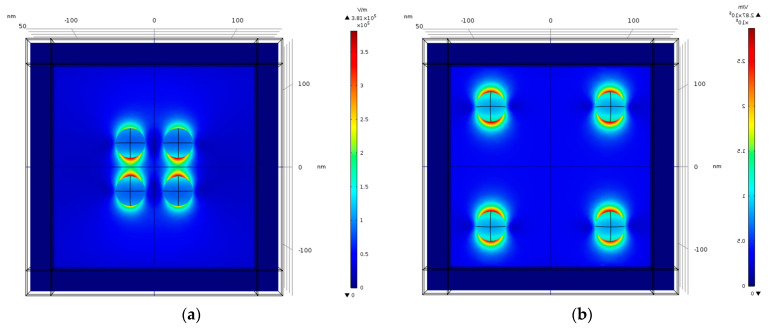
Simulation of separated hemi-spheroid nano-particles. (**a**) Separation of 20 nm; (**b**) separation of 110 nm. The width of physical geometry W = 250 nm. The electric field polarization is in the Y direction, therefore the localized surface plasmon is excited mostly in that direction. There is less interaction between nano-particles separated in the X direction.

**Figure 24:**
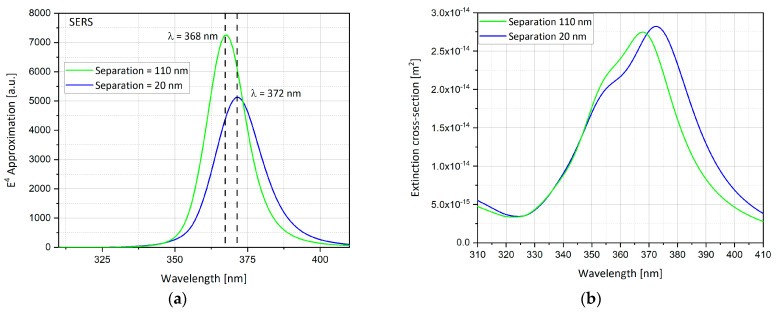
Simulation of four separated hemi-spheroid particles (separation = 20 nm, 110 nm). The width of physical geometry W = 250 nm. (**a**) *E*^4^ approximation; (**b**) extinction cross-section.

These separations are compared in Figure 23 and Figure 24. In these simulations, the width of the physical geometry W = 250 nm.

As depicted in Figure 24a, separation of 110 nm produces a higher peak than separation of 20 nm. The nano-particles (Figure 23a) are very close, which cause plasmons to interact with each other, therefore the mutual dipole suppression effect decreases the electric field enhancement. However, as the separation gets bigger this plasmon interaction gets weaker (Figure 23b), therefore mutual dipole enhancement produces higher electric field enhancement and reaches an optimal separation around 110 nm. The peaks of the field enhancement are produced at different wavelengths. The wavelength for the resonant condition is red-shifted as the gap between nano-particles gets smaller. Figure 24b shows that the extinction cross-section peak for the separation of 110 nm is lower than the extinction cross-section peak for the separation of 20 nm. However, the peaks are produced at different wavelengths and the difference between them is small, therefore the optimal separation is 110 nm, which also produces higher electric field enhancement as shown in Figure 23a.

A pixel design was devised, based on a finite array of hemi-ellipsoidal silver nanostructures of radius 20 nm and an aspect ratio, A.R. = 2.00, on a silicon substrate. An initial study was conducted to determine the optimal number of structures per pixel, or equivalently—for fixed pixel dimension—the optimal distance between them. The initial design chosen comprises 121 structures arranged in a finite square array of dimension 1.1 µm with 11 structures in each direction—a separation of 110 nm.

Further validation studies are necessary, comparing the results of the simulation to analytical results [1] for a few simple geometries such as the sphere and ellipsoid. A comparison of the performance of cavities, and particles of the same shape, will be examined. The particles and protrusions are expected to show better enhancement than the corresponding cavities. The cavity–particle duality will be verified next. The prediction of the ESA for spherical shells will be compared to the simulation for both static and propagating fields; the shape itself will then be optimized. The hemi-spheroids used in the design possess a sharp edge along the bottom face. The significance of the resulting singular field in particular, and the deviation of the performance from ideal spheres and ellipsoids in general, must be examined.

## 5. Preliminary Experimental Results

### Protrusions and Cavities Arrays Fabrication and Structural Characterization

Following the above numerical and analytical analyses, specifying the definition of the optimal material and geometry of the individual tip-probe of the pixels array, preliminary experimental results were performed. Several arrays of protrusions and cavities were fabricated. The arrays were manufactured using a Focused Ion Beam (FIB) milling equipment, at Bar-Ilan university Institute for Nanotechnology and Advanced materials (BINA). Integrated SEM served to characterize the fabrication and to monitor the quality of the samples. It should be noted that due to the COVID-19 world pandemic, we dealt with major limitations and restrictions on regular laboratory work. A series of additional experiments are scheduled to be held in the near future.

**Figure 25:**
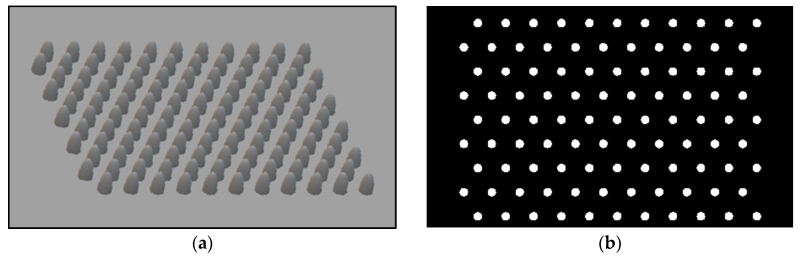

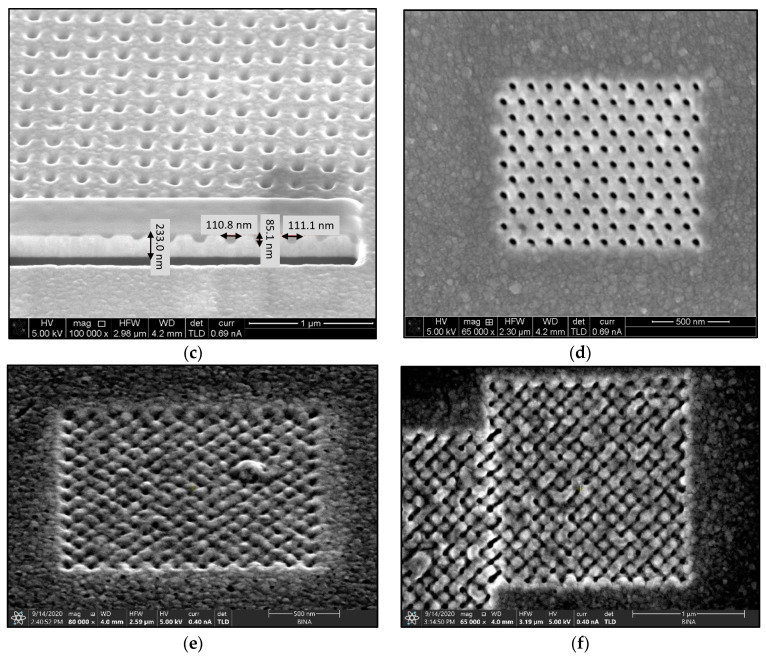
SEM pictures of preliminary samples fabricated with FIB technique. (**a**) Simulation mask of an array of tips before the fabrication of the protrusions; (**b**) simulation mask of holes array before the fabrication of cavities. (**c**) Nano-holes square lattice of cavities; (**d**) nano-holes hexagonal lattice of cavities; (**e**) nano-protrusions array using FIB; (**f**) nano-protrusion double-array fabrication using FIB.

The architecture and design steps required extensive work of optimization until reaching the final array, since there is no significance to a single stand-alone tip-probe for these scanning applications. The following figure presents the design of arrays and masks of nano-cones (Figure 25a) and nano-holes (Figure 25b), the fabrication of arrays of cavities (Figure 25c,d), and of protrusions (Figure 25e,f).

While the protrusions are difficult to obtain, the cavities were obtained in a much more straightforward resolution. Looking at the dimensions of the pixel, and in particular at the total array active area (white space), one can obtain a matrix of width × height = 1300 nm × 1080 nm. In fact, the active area consists of an arrangement of 11 × 11 nanostructures. In a preliminary configuration, the structures are depressions (i.e., open cavities), to be drilled into the silver layer. The opening is circular of radius r = 20 nm. The separation distance between structure centers is 120 nm, so the separation between the structure edges remains 80 nm. Regarding the pixel depth and repetition, the following dimensions were chosen: 10 pixels separated by at least 5 to 10 µm for a good separation in an optical microscope. The structure of the first pixel should have a depth of 20 nm, i.e., it should be semi-spherical. The other pixels should be of increasing depth until a maximum depth of 120 nm. Recording the current and time used for each pattern was crucial in order to determine the plasmonic properties as a function of the ion dose. Pictures of the arrays design and of the preliminary results are presented in Figure 25.

## 6. Discussion

### 6.1. The Nano-Particles Geometry

The simulations from previous sections provide an insight to what is the optimal nano-particle geometry. Analyzing Figure 12 and Table 3, one can observe that the hemi-spheroid geometry is the most optimal one for obtaining the highest SERS EF. Looking at Figure 9b, it seems clear that the ellipsoidal cavity provides the highest extinction cross-section. Moreover, the ellipsoidal rod and the double nano-cone simulation provide excellent insight into the effect off polarization of the electric field on specific geometries. The nano-cone simulation produced very low SERS EF, because the polarization of the electric field was not aligned with the tip of the cone. This outcome was verified in the double nano-cone simulation which produced higher SERS EF, because the polarization was aligned with the tips of the double nano-cone geometry.

More research is required in order to ascertain what the optimal eccentricity of the hemi-spheroid should be. Moreover, combining different geometries such as hemi-spheroid with a nano-cone could provide a better SERS EF, or even act as another tool to tune the resonant frequency, as was demonstrated in the nano-shells geometry. For the first generation of a TERS device, the hemi-spheroid geometry is most certainly going to be a good starting point for device measurements, characterization and advancing research in this direction.

### 6.2. Particles Material and Nano-Shells Tuning

The simulations from Section 4.13 provide further insight into which material should be used for the nano-particles. Silver shows great promise in the wavelength region of 325–495 nm (Figure 14). Silver produces the highest SERS EF and extinction cross-section, but aluminum could be used in the region 50–325 nm which could be more optimal than the UV region.

The simulations from Section 4.14 provide a better understanding of the geometry of the nano-shells and its advantages in tuning the resonant wavelength, Moreover, Figure 16 demonstrates that as the nano-shell thickness decreases, the resonant wavelength increases and the SERS EF becomes much larger. The nano-shells geometry could be used by combining different materials such as gold and silver to receive a different resonant condition and a better chemical reaction to the solution that will be present near the nanostructures. Gold is known to be very stable in solution, whereas silver is very unstable. Further research should be done to determine the best combination of materials in the nano-shells geometry.

### 6.3. SERS/TERS Nano-Particles Separation

The simulations from Section 4.15 provide further insight into the effect of the mutual influence of multiple nanostructures. In the SERS/TERS simulation, a square pixel geometry was used with four hemi-spheroid nano-particles with eccentricity of e = 0.866 as shown in Figure 19. Figure 20 presents the mutual influence of the four nano-particle effects on the SERS EF. The separation between the nano-particles affects the SERS EF, therefore an optimal separation between the nano-particles was researched. In Figure 20, Figure 21, Figure 22, Figure 23 and Figure 24, it appears that the separation of 110 nm between the nano-particles is the most optimal for getting the highest SERS EF. More research is required in order to determine how many nano-particles there should be per pixel and to determine the size of each pixel. Moreover, the hexagon pixel geometry should be researched in order to determine the optimal pixel shape (square or hexagon).

## 7. Conclusions

In this article, several directions were investigated with the final purpose of a full-scale production of a Tip-Enhanced Raman Scattering (TERS) device. Spatial distribution of enhanced electric field around metal tip for TERS was reported. The investigations focused on the effect of shape (hemi-sphere, hemispheroid, ellipsoidal cavity, ellipsoidal rod, nano-cone), and the effect of material (Ag, Au, Al) on enhancement, as well as the effect of excitation wavelengths on the electric field. The background of theoretical physics with its implementation in the simulations, yields a successful conclusion to the geometries that were analyzed. From the results section, it appears that the recommendation is for hemi-spheroid geometry for the nano-particles, and its eccentricity will be a significant parameter in the characterization of the next generation of TERS devices towards production feasibility. When analyzing the material options, silver is recommended. The use of nano-shells is a viable option for tuning the resonant wavelength of the device. To fully characterize a TERS structure, research should be directed toward combining different kinds of nano-particles geometries and their arrangement in the SERS array, in square or hexagon geometry, as previously started [44]. SERS array in hexagon geometry should be examined as well, in order to determine which geometry (square or hexagon) is better suited for enhanced performance. Additionally, optimization for separation of nano-particles and density of particles in each pixel must be performed in order to make the device’s SERS EF in optimal conditions. For the next generation of TERS imagers, a beyond *E*^4^ approximation approach must be examined in order to simulate a near-field Raman effect dipole emitter in the nano-structure vicinity. By examination of the *E*^4^ approximation and of the extinction cross-section in various geometries, the device was accurately modeled analytically and numerically.

## 8. Patents

This research is the basis for several future patents.

## Figures and Tables

**Table 1:** Parameters for all geometries and parametric sweeps.

Name	Expression	Description
W	150 nm, 250 nm, 450 nm	Width of physical geometry
t_pml	30 nm	Perfectly Matched Layer (PML) thickness
h_air	80 nm	Air domain height
h_subs	50 nm	Substrate domain height
T	1.433–8.303 nm	Nano-shell thickness (using Δ = 0.2, 0.4, 0.6, 0.8)
R	20 nm	Nano-particle radius
A	20 nm	Ellipsoid x semi axis
B	20 nm	Ellipsoid y semi axis
C	40 nm	Ellipsoid z semi axis
E	0.866	Eccentricity
Na	1	Air refractive index
Phi	0, π/2	Azimuthal angle of incidence
θ	0, π/6, π/4, π/3	Polar angle of incident field
*I*_0_	10^6^ W/m^2^	Intensity of incident field
P	I_0_w^2^cos(θ)	Port power
Sep	5–315 nm	Separation between particles

**Table 2:** Variables and functions used during the simulations.

Name	Expression	Unit	Description	Domain
ewfd.Ex	0	V/m	X direction electric field	PML Domain
ewfd.Ey	0	V/m	Y direction electric field	PML Domain
ewfd.Ez	0	V/m	Z direction electric field	PML Domain
E0x	-sin(phi)		Amplitude of Ex in X	Port 1,2
E0y	cos(phi)		Amplitude of Ey in Y	Port 1,2
intop_surf			Surface integral	nano-particle surface
intop_vol			Volume integral	nano-particle volume
nrelPoav	n_x_ * ewfd2.relPoavx + n_y_ * ewfd2.relPoavy + n_z_ * ewfd2.relPoavz	W/m^2^	Relative normal Poynting flux	Entire model
Sigma_sc	intop_surf(nrelPoav)/*I*_0_	m^2^	scattering cross-section	Entire model
Sigma_abs	intop_vol(ewfd2.Qh)/*I*_0_	m^2^	absorption cross-section	Entire model
Sigma_ext	Sigma_sc+ Sigma_abs	m^2^	extinction cross-section	Entire model

**Table 3:** Comparison table of the studied particle’s shapes based on *E*^4^ approximation.

Studied Shape	Checked Polar or Azimuth Angles	λ Peak Enhancement	*E*^4^ at Peak	Comments
Hemi-sphere	θ = 0, π/6, π/4, π/3	λ = 368 nm	2460	● Largest extinction cross-section at θ = 0
Spheroidal cavity	θ = 0, π/6, π/4, π/3	λ = 372 nm	1865	● Largest extinction cross-section at θ = 0. ● Easiest shape using Focused Ion Beam (FIB).
Hemi-spheroid	θ = 0, π/6, π/4, π/3	λ = 368 nm	7310	● Largest extinction cross-section at θ = 0. ● Eccentricity of 0.866. ● Highest field enhancement.
Nano-cone	θ = 0, π/6, π/4, π/3	λ = 357nm	208	● Largest extinction cross-section at θ = 0. ● Nano-cone surface area = hemi-sphere’s area.
Ellipsoidal cavity	θ = 0, π/6, π/3	λ = 375nm	6832	● Largest extinction cross-section at θ = 0. ● Easiest feasible shape using FIB. ● Highest extinction cross-section.
Ellipsoidal rod	θ=0, φ=0, π/2	λ = 378nm	7038	● Largest peak at φ = 0. ● Sensitive to electric field polarization
Double nano-cone	θ=0, φ=0, π/2	λ = 375nm	5446	● Largest peak at φ = $\frac{\pi }{2}.\;$ ● Sensitive to electric field polarization
